# EEG measures of sensorimotor processing and their development are abnormal in children with isolated dystonia and dystonic cerebral palsy

**DOI:** 10.1016/j.nicl.2021.102569

**Published:** 2021-01-19

**Authors:** Verity M McClelland, Petra Fischer, Eleonora Foddai, Sofia Dall'Orso, Etienne Burdet, Peter Brown, Jean-Pierre Lin

**Affiliations:** a Department of Basic and Clinical Neuroscience, Institute of Psychiatry, Psychology and Neuroscience, King’s College London, London SE5 9RX, United Kingdom; b Medical Research Council Brain Network Dynamics Unit and Nuffield, Department of Clinical Neurosciences, University of Oxford, Oxford OX1 3TH, United Kingdom; c Department of Biomedical Engineering and Human Robotics, Imperial College London, London SW7 2AZ, United Kingdom; d Children’s Neurosciences Department, Evelina London Children’s Hospital, Guy’s and St Thomas NHS Foundation Trust, London SE1 7EH, United Kingdom

**Keywords:** AMPS, Assessment of Motor and Process Skills, BFMDRS-m, Burke-Fahn-Marsden Dystonia Rating Scale motor score, CP, Cerebral Palsy, DBS, Deep Brain Stimulation, EEG, electroencephalogram, EMG, electromyogram, ERD, event-related desynchronization, ERS, event-related synchronisation, MEG, magnetoencephalogram, SEP, Somatosensory Evoked Potential, StretchEP, Stretch Evoked Potential, TTL, transistor to transistor logic, Dystonia, Dystonic cerebral palsy, Event-related synchronisation, Event-related desynchronisation, Mu modulation, Sensorimotor integration

## Abstract

Dystonia is a disorder of sensorimotor integration associated with abnormal oscillatory activity within the basal ganglia-thalamo-cortical networks. Event-related changes in spectral EEG activity reflect cortical processing but are sparsely investigated in relation to sensorimotor processing in dystonia. This study investigates modulation of sensorimotor cortex EEG activity in response to a proprioceptive stimulus in children with dystonia and dystonic cerebral palsy (CP).

Proprioceptive stimuli, comprising brief stretches of the wrist flexors, were delivered via a robotic wrist interface to 30 young people with dystonia (20 isolated genetic/idiopathic and 10 dystonic CP) and 22 controls (mean age 12.7 years). Scalp EEG was recorded using the 10–20 international system and the relative change in post-stimulus power with respect to baseline was calculated for the alpha (8–12 Hz) and beta (14–30 Hz) frequency bands.

A clear developmental profile in event-related spectral changes was seen in controls. Controls showed a prominent early alpha/mu band event-related desynchronisation (ERD) followed by an event-related synchronisation (ERS) over the contralateral sensorimotor cortex following movement of either hand. The alpha ERD was significantly smaller in the dystonia groups for both dominant and non-dominant hand movement (ANCOVA across the 3 groups with age as covariate: dominant hand F(2,47) = 4.45 p = 0.017; non-dominant hand F(2,42) = 9.397 p < 0.001. Alpha ERS was significantly smaller in dystonia for the dominant hand (ANCOVA F(2,47) = 7.786 p = 0.001). There was no significant difference in ERD or ERS between genetic/idiopathic dystonia and dystonic CP.

**Conclusion:**

Modulation of alpha/mu activity by a proprioceptive stimulus is reduced in dystonia, demonstrating a developmental abnormality of sensorimotor processing which is common to isolated genetic/idiopathic and acquired dystonia/dystonic CP.

## Highlights

• Modulation of alpha/mu activity by proprioceptive stimuli is reduced in dystonia.
• Changes in mu modulation seen in typical development are diminished in dystonia.
• These abnormalities are common to genetic/idiopathic dystonias and dystonic CP.

## 1 Introduction

Dystonia is a network disorder involving dysfunction within the basal ganglia, cortex, cerebellum or their inter-connections as part of the sensorimotor network (Corp et al., 2019, Neychev et al., 2011). Abnormal sensory processing has been demonstrated in neurophysiological (Tinazzi et al., 2000, Frasson et al., 2001) and imaging studies (Nelson et al., 2009), supporting the concept that dystonia is a disorder of sensorimotor integration, in which distorted processing of sensory information leads to excessive/uncontrolled motor outputs (Tinazzi et al., 2000).

Although many studies demonstrate abnormal sensory processing in dystonia (Frasson et al., 2001, Tinazzi et al., 2003, Desrochers et al., 2019, Avanzino et al., 2015), most focus on adults with isolated genetic or idiopathic dystonias and few studies have investigated sensory processing in acquired dystonia or in dystonia during childhood (McClelland, 2017), when sensorimotor development is still ongoing. This important aspect of dystonia pathophysiology is often neglected in dystonia research (McClelland, 2017), despite the fact that many isolated genetic dystonias present in early-mid childhood and many acquired dystonias arise from perinatal onset lesions (e.g. dystonic cerebral palsy (CP)). Understanding the common and divergent features between these different types of dystonia, and their developmental aspects, is essential for creating a more individualised approach to therapy and addressing differential outcomes from neuromodulation such as pallidal deep brain stimulation (DBS).

A recent paediatric study in over 100 children with dystonia reported that a large proportion (47%) of the cohort had abnormal sensory evoked potentials (SEPs), demonstrating some degree of disruption of function in primary sensory pathways (McClelland et al., 2018). Interestingly, the majority of the abnormalities were seen in patients with acquired dystonia. This finding is in keeping with the network concept, with these individuals showing evidence of disruption at a different node of the sensorimotor network from those with isolated genetic dystonias, in whom SEPs were normal. However, a large proportion of children with acquired dystonia still had normal SEPs, suggesting that any abnormality of the sensorimotor network in these individuals must be further “downstream” than the initial arrival of afferent information at the cortex (McClelland et al., 2018). Further investigation of sensory processing in this group is therefore warranted.

Event-related changes in spectral EEG activity are considered to reflect cortical processing (Pfurtscheller, 2001), but are sparsely investigated in dystonia. One study reported abnormal event-related spectral changes during an active motor task in patients with hemi-dystonia (Kukke et al., 2015), whilst another recent study demonstrated abnormal modulation of corticomuscular coherence during a motor task in children with genetic/idiopathic and acquired dystonia (McClelland et al., 2020).

The current study investigates sensory processing in children with isolated genetic/idiopathic dystonias and dystonic CP by measuring changes in cortical oscillatory activity in response to a proprioceptive stimulus. We focus on the alpha/mu spectral frequency band (8–12 Hz) since desynchronised activity in this range is typically seen over sensorimotor cortex during passive movement or somatosensory stimulation (Pfurtscheller, 2001, Pineda, 2005). Additionally, this band coincides in part with the 4–12 Hz range in which abnormal cortical, sub-cortical and intermuscular oscillatory activity has been reported in genetic/idiopathic dystonia (Neumann et al., 2012, Sharott et al., 2008, Barow et al., 2014, Miocinovic et al., 2017) (McClelland et al., 2020, Sharott et al., 2008, Neumann et al., 2015, Doldersum et al., 2019). We thus test the hypothesis that levels of alpha ERD and ERS over sensorimotor cortex in response to the proprioceptive stimulus will differ between controls, isolated genetic/idiopathic dystonia and dystonic CP.

## 2 Materials and methods

### 2.1 Ethical approval

Ethical approval was obtained from the London-Harrow National Research Ethics Committee, London, UK (17/LO/0439). Informed written consent was obtained from the participant or, if under 16 years old, from parents with assent from the child. The studies were conducted in accordance with the declaration of Helsinki.

### 2.2 Subjects and experimental arrangement

The studies were performed on 35 young people with dystonia, recruited from the Complex Motor Disorders Service at Evelina London Children’s Hospital, and on 22 typically developing children/young people, with no history of neurological disorders. Age-range was 5–21 years. The diagnosis and classification of dystonia was confirmed by a consultant paediatric neurologist with specialist expertise in movement disorders (JPL), and are summarised in Table 1. Data from 5 patients were excluded (see Results for details). Of the remaining 30 participants with dystonia, 19 had bilateral Globus Pallidus internus DBS in situ. For these individuals, the DBS was on for the duration of the recording, with stimulation frequency of 130–200 Hz. Severity of dystonia was assessed by the specialist clinical and therapy team, using the motor score of the Burke-Fahn-Marsden Dystonia Rating Scale (BFMDRS-m). Additionally, some of the patients were assessed with the AMPS (Assessment of Motor and Process Skills) score.

**Table 1:** Clinical details of participants with dystonia.

	Classifi-cation	Aetiology	Phenotype	Age group (yrs)	Hand dom-inance	Age of dystonia onset (years)	Cranial MRI	DBS	Duration between Diagnosis and DBS (years)	Duration between DBS and Study (years)	GMFCS (Baseline)	BFMDRS-movement score (at time of study)	BFMDRS Disability score (at time of study)
1	Isolated genetic	DYT1	Onset R arm progressing to generalised dystonia, frequent falls	5–9	R	7.00	Normal	N			4	57	11
2	Isolated genetic	DYT1	Generalised dystonia	5–9	R	5.00	Normal	N			3	47	12
3	Isolated genetic	DYT1	Onset L hand, progressing to generalised dystonia	10–14	R	8.00	Normal	N			4	52	14
4	Isolated genetic	DYT1	Onset R hand progressing to generalised dystonia	10–14	R	9.00		N					
5	Isolated genetic	DYT1	Craniocervical onset progressing to generalised dystonia	15–21	R	8.50	Normal	Y	1.94	0.50	3	38	11
6	Isolated genetic	DYT1	Generalised dystonia, particularly with dystonic posturing in left foot and right hand/wrist	10–14	R	10.00	Normal	Y	3.60	4.62	1	12	4
7	Isolated genetic	DYT11	Dystonia myoclonus	10–14	R	1.50		N			1	34.5	13
8	Isolated genetic	DYT11	Dystonia myoclonus. Onset with falls	15–21	R	4.00	Normal	Y	5.83	6.67	2	20	6
9	Isolated genetic	DYT11	Dystonia myoclonus with possible benign hereditary chorea	15–21	L	3.00	No basal ganglia abnormalityIncidental Chiari type 1 malformation	Y	15.05	0.82	1	38.5	5
10	Isolated genetic	DYT11	Dystonia myoclonus	15–21	L	1.50	Normal	Y	13.59	6.73	1	16.5	2
11	Isolated genetic	DYT6 (THAP1)	Dystonia onset initially right foot turning in and frequent falls. Progressed to generalised dystonia.	10–14	R	4.50	Subtle susceptibility related signal loss within globus pallidus	Y	5.19	1.15	4	53	15
12	Isolated genetic	DYT6 (THAP1)	Onset with left leg turning out, progressed to generalised dystonia.	10–14	L	6.00	Normal	Y	1.46	7.48	4		
13	Isolated genetic	KMT2B	Generalised movement disorder with sustained postures and hyperkinetic movements.	5–9	R	1.40	Normal	N			2	46.5	11
14	Isolated genetic	KMT2B	Progressive ascending asymmetric dystonia (Right more than left)	15–21	R	3.00	Subtle gliotic change in posterior putamina	N			1	66	9
15	Isolated genetic	KMT2B	Generalised dystonia onset age 2yrs with toe walking.	5–9	R	2.25	Slightly increased susceptibility in pallidum	Y	5.42	0.82	2	80.5	19
16	Isolated genetic	KMT2B	Progressive movement disorders with dystonia, dystonic tremor and some Parkinsonian features (festination, hypomimia)	10–14	R	2.00	Slightly increased susceptibility in pallidum	Y	10.36	1.54	4	72	21
17	Idiopathic	Idiopathic	Primary dystonia with segmental involvement involving both feet and legs. Very little upper limb involvement.	10–14	R	4.50	Normal	N			2	24	2
18	Idiopathic	Idiopathic	Onset generalised dystonia age 20 months, initially limping on right side, progressed to generalised dystonia.	5–9	R	1.75	Normal	Y	1.32	4.15	5	45	18
19	Idiopathic	Idiopathic	Progressive movement disorder with elements of dystonia and action tremor.	15–21	R	0.60	Normal	Y	7.77	7.20	2	37	9
20	Idiopathic	Idiopathic	Generalised dystonia with tremor and myoclonus	15–21	L	0.50	Normal	Y	12.42	5.14	1	37	8
21	Acquired	CP HIE	Generalised dystonia affecting principally left arm and both legs. Typical action dystonia	5–9	R	N/A	Increased T2 signal in posterior putamen	N			2	60	13
22	Acquired	CP HIE	Severe action dependent dystonic choreoathetosis with severe dystonic tremor.	10–14	L	3.00	Subtle posterior Putaminal and ventrolateral thalamic gliosis, in keeping with term HIE.	Y	5.16	4.31	3	40	9
23	Acquired	CP HIE	Generalised dystonia and athetosis	15–21	R	0.25	Putaminal gliosis in keeping with term HIE	Y	13.13	4.20	2	55	10
24	Acquired	CP HIE	Generalised dystonia with action specific dystonic tremor, more marked for manual activities than gross motor skills. Dystonia dysarthria.	15–21	L	0.50	Subtle deep white matter change	Y	16.37	1.09	1	27	10
25	Acquired	CP HIE	Dystonia and chorea, predominantly right sided, upper limb > lower limb.	15–21	L	0.75	Left putaminal gliosis; milder change left ventrolateral thalamus. In keeping with HIE but asymmetrical (predominantly left).	Y	16.57	1.65	1	42.5	9
26	Acquired	CP HIE	Generalised dystonia	15–21	R	0.50	Bilateral putaminal and thalamic gliosis, in keeping with term HIE	Y	12.38	6.18	3	60.5	18
27	Acquired	CP Prem	Mixed movement disorder with generalised dystonia and chorea. Also stereotypies.	5–9	L	0.50		N					
28	Acquired	CP Prem	Mixed dystonia and spasticity	15–21	L	0.50		N					
29	Acquired	CP Prem + kernicterus	Generalised dystonic choreoathetosis	10–14	R	0.10	Deep periventricular white matter abnormality with additional gliosis of globi pallidi	Y	10.78	0.75	2–3	86	22
30	Acquired	CP Prem + kernicterus	Generalised dystonia-dyskinesia	10–14	L	0.50	Paucity of deep white matter volume	Y	10.62	3.74	2	62	15

**Fig. 1:**
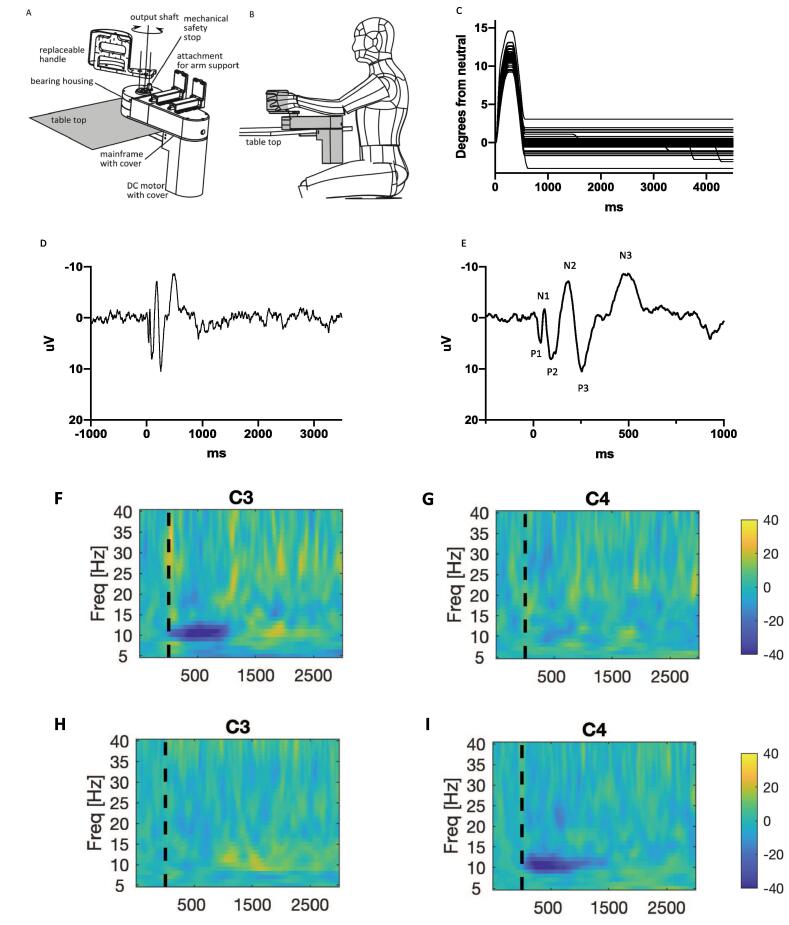
**Experimental paradigm and sample data** Top row: Experimental set-up. A: Design overview of Portable Hi5 interface, which can be used with various handles and end effectors. B: User interacting with Hi5 attached to a table-top. Line drawings kindly provided by Ildar Farkhatdinov from (Sakellariou et al., 2020). C: Movement profile of wrist extension in degrees from neutral position over time (ms) in a single subject aged 8 years old. Each line shows the movement profile for an individual trial (N = 153 trials). D: Cortical evoked potential recorded over contralateral sensorimotor cortex in same subject as part C (in this case over C3 electrode during right wrist movement). Figure shows average of 139 epochs. E: Same as part D but with shorter time-scale to show the three positive and three negative primary components of the stretchEP. F-I. Time frequency plots from a single subject aged 6 years old. x-axis shows time in ms after the stimulus (dashed vertical line), y-axis shows frequency, colour scale shows relative power (%) at each frequency with respect to the pre-stimulus period such that dark blue indicates event-related desynchronisation and yellow-orange indicates event-related synchronization. F-G show results from right hand movement recorded over C3 (hemisphere contralateral to stretch) and C4 (hemisphere ipsilateral to stretch) respectively. H-I show results from left hand movement recorded over C3 (hemisphere ipsilateral to stretch) and C4 (hemisphere contralateral to stretch) respectively. (For interpretation of the references to colour in this figure legend, the reader is referred to the web version of this article.)

Participants were seated comfortably at a table with their arm positioned in the armrest of a robotic wrist interface (named the “portable Hi5”), designed to produce controlled passive wrist extension movements. The portable Hi5 was modified from a previous study investigating tactile sensation in adults (Wilhelm et al., 2016) and is in keeping with a similar device used for the investigation of brain development in neonates (Allievi et al., 2013). It was modified to accommodate the current protocol (Fig. 1A-B). The same equipment and protocol were applied in a companion study investigating neuronal connectivity in a sub-set of the patients (Sakellariou et al., 2020). The portable Hi5 interface is designed to be mounted easily on a table. The forearm is positioned and supported in mid-supination, parallel to the table, while the hand is strapped to a customised hand-piece with the wrist joint aligned with the pivot point. The participants were instructed to relax their arm as much as possible and to allow the Hi5 to make the movements. (Inability to place and keep the wrist in the device was an exclusion criterion). A trial set of epochs was run, to allow the participant to become familiar with the feeling of the wrist movement and to relax. Participants were allowed to listen to music or watch a movie of their choice during the study in order to help them relax and to prevent them from focussing on their hands. Two mirror-image hand-pieces were designed to suit the hand of the participants in the age-range of the study and could be interchanged to allow movements to be performed for either the right or left hand. Both hands were tested in each subject, with semi-random order as to which was tested first. Hand dominance was recorded, according to self and parental report. The hand-piece was moved ad hoc by a motor to produce a brief passive wrist extension, followed by return to neutral, thus providing a brief stretch of the wrist flexors. The movement followed the profile of the half period of a sine wave (1 Hz), with rise time of 240 ms and a target of 12° from the neutral position. Across the group, the actual excursion of the wrist was in the range 5-15° due to intrinsic mechanical factors and varying resistance of the subject’s hand (Fig. 1C – movement profile). This position information was recorded throughout the study and analysed off-line. Stimulus control, monitoring and synchronization between stimuli and EEG recordings was achieved through a custom code developed by the authors in the LabVIEW software environment (National Instruments, Austin, TX, USA).

The Hi5 device was programmed to repeat the wrist extension movement at pseudorandom intervals of 5 s +/- 0.5 s to prevent anticipation. Movements were performed in blocks of 10 and the participant was given the opportunity to rest between blocks if required. Up to 160 wrist extension movements were recorded for each hand. The movement of the hand-piece was measured via a position sensor incorporated into the device and was recorded at 1000 Hz. Additionally, the LabVIEW environment provided visual feedback so that the researcher could monitor the hand position throughout the experiment. A TTL (transistor-transistor logic) pulse was passed from the Hi5 interface to the EEG recording system to synchronise the movement onset with the simultaneous EEG and EMG recordings.

### 2.3 Recordings

Scalp EEG was recorded with Ag/AgCl electrodes sited according to the 10–20 international system at Fp1, Fp2, F7, F3, Fz, F4, F8, Fc5, FC1, FC2, FC6, T3/C5, C3, Cz, C4, T4/C6, CP5, CP1, CP2, CP6, P7/T5, P3, Pz, P4, P8/T6, Oz, using FCz as a reference and the ground electrode at AFz. Electrodes were applied to the scalp using conductive paste (Ten20, Weaver and Company) and impedances were maintained below 10 kOhm. Surface EMG was recorded using self-adhesive electrodes (Neuroline 700, Ambu) applied to forearm flexors and extensors in a belly-tendon montage. EEG and EMG were amplified, filtered between DC and 500 Hz and sampled at 2500 Hz using a BrainVision system (BrainAmp MR Plus).

### 2.4 Offline analysis

The profile of the wrist movements was analysed in Matlab. Epochs with an abnormal wrist movement profile were excluded (i.e. those epochs with peak excursion of < 5° or in which the position during the resting period strayed 5 or more degrees from neutral in either direction). A lag time of 3–30 ms was observed between the TTL pulse and the actual movement onset, reflecting slight differences in inertia of the wrist joint between subjects. To account for this, the true movement onset was defined as the sample point at which a continuous increase in joint position, as recorded by the Hi5 interface, was observed over 5 consecutive sample points. The EEG markers based on the TTL pulse were then amended to reflect this true movement onset for each epoch, using the Brain Vision Analyser “Edit Markers” function.

EEG data were pre-processed in BrainVision Analyser. Recordings were down-sampled to 1000 Hz (to match position data sampling) and movement onset markers were edited as above. A low-pass filter with a cut-off of 70 Hz was applied offline in all subjects to filter DBS artefacts from the EEG data (see also Discussion). A 50 Hz notch filter was also applied to suppress line noise. Data were then segmented into epochs based on the corrected movement onset markers, comprising 1 s pre-stimulus and 3.5 s post-stimulus. Artefact rejection was performed manually to remove those epochs with inadequate wrist movement profile and those contaminated by excessive movement or eye blink artefacts. Following artefact rejection, the mean number of epochs per hand was 107 (95% CI 102 – 112). These remaining epochs were then baseline corrected in BrainVision Analyser, by calculating the average of the data in the pre-stimulus period and subtracting this from each data point in the post-stimulus period. This was done for each channel. The baseline-corrected epochs were then averaged to reveal the Evoked Potential/Event-related Potential (ERP: Fig. 1D-E) for each hand in each subject. The peaks of the primary ERP components were identified semi-automatically using the Brain Vision Analyser Peak Detection function and peak-to-peak amplitudes were calculated. EEG data were exported for further analysis in the frequency domain using Matlab software (FieldTrip toolbox) customised by the authors.

### 2.5 Time frequency analysis

**Fig. 2:**
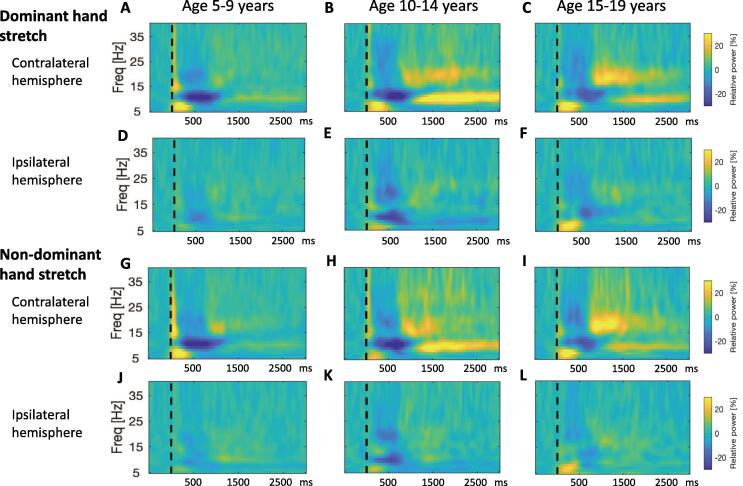
**Developmental Sequence of event-related changes in EEG power in relation to a proprioceptive stimulus in typically developing children.** Pooled time–frequency plots for control subjects, grouped by age. Left column: Young age group (5–9 years, n = 10), middle column: Intermediate age group (10–14 years, n = 6), right column: Older age group (15–21 years, n = 6). x-axis shows time in ms after the stimulus (dashed vertical line), y-axis shows frequency, colour scale shows relative power at each frequency with respect to the pre-stimulus period, such that dark blue indicates event-related desynchronisation and yellow-orange indicates event-related synchronization. A-C: Response over contralateral hemisphere to dominant hand stretch (ie right sensorimotor cortex for left hand movement, left sensorimotor cortex for right hand movement). D-F: Response over ipsilateral hemisphere to dominant hand stretch. G-I: Response over contralateral hemisphere to non-dominant hand stretch. J-L Response over ipsilateral hemisphere to non-dominant hand stretch. (Note the sharp increase in power with respect to baseline at time zero, extending up to 40 Hz, and the brief, early increase in theta range power from 0 to 300 ms are likely to reflect movement artefact and a contribution from the stretchEP, respectively). (For interpretation of the references to colour in this figure legend, the reader is referred to the web version of this article.)

**Fig. 3:**
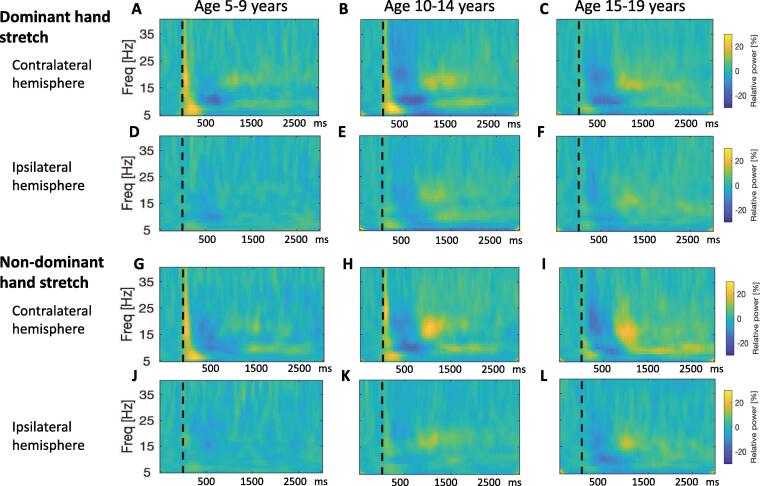
**Developmental Sequence of event-related changes in EEG power in relation to a proprioceptive stimulus in children with dystonia.** Pooled time–frequency plots for individuals with dystonia, grouped by age: Left column: Young age group (5–9 years, n = 7), middle column: Intermediate age group (10–14 years, n = 11), right column: Older age group (15–21 years, n = 12). x-axis shows time in ms after the stimulus (dashed vertical line), y-axis shows frequency, colour scale shows relative power at each frequency with respect to the pre-stimulus period, such that dark blue indicates event-related desynchronisation and yellow-orange indicates event-related synchronization. A-C: Response over contralateral hemisphere to dominant hand stretch (ie right sensorimotor cortex for left hand movement, left sensorimotor cortex for right hand movement). D-F: Response over ipsilateral hemisphere to dominant hand stretch. G-I: Response over contralateral hemisphere to non-dominant hand stretch. J-L Response over ipsilateral hemisphere to non-dominant hand stretch. (Note for non-dominant hand, n = 9 for both intermediate and older age groups). (For interpretation of the references to colour in this figure legend, the reader is referred to the web version of this article.)

EEG power for frequencies from 5 to 40 Hz was calculated in 1 Hz bins using the continuous Morlet wavelet transform (*fieldtrip*-function *ft_freqanalysis*, RRID:SCR_004849 (Oostenveld et al., 2011)). The number of wavelet-cycles was 8. The normalised resting power in the theta (3–7 Hz), alpha/mu (8–12 Hz) and beta (14–30 Hz) frequency bands was calculated for each individual by dividing the power in each frequency bin by the total power in the 1–70 Hz range during the baseline/pre-stimulus period (-500 ms to 0 ms). Event-related changes in spectral activity were calculated for each individual by computing, for each frequency bin, the change in power with respect to the stimulus (time zero) as the percentage increase or decrease from the mean power in the baseline period. The frequency and time point at which the maximum event-related desynchronization (ERD) in the alpha/mu range (8–12 Hz) occurred was also identified for each individual. Analysis was performed separately for the dominant and non-dominant hands. The event-related changes were averaged across all epochs for a given hand movement in each individual. Individual time–frequency plots were produced for each hand, showing changes over contralateral and ipsilateral hemispheres (Fig. 1F-I). For group analyses (Fig. 2, Fig. 3), relative power was averaged across subjects, separately for dominant and non-dominant hands, to show contralateral EEG change (right sensorimotor cortex for left hand movement, left sensorimotor cortex for right hand movement) or ipsilateral EEG change.

**Fig. 4:**
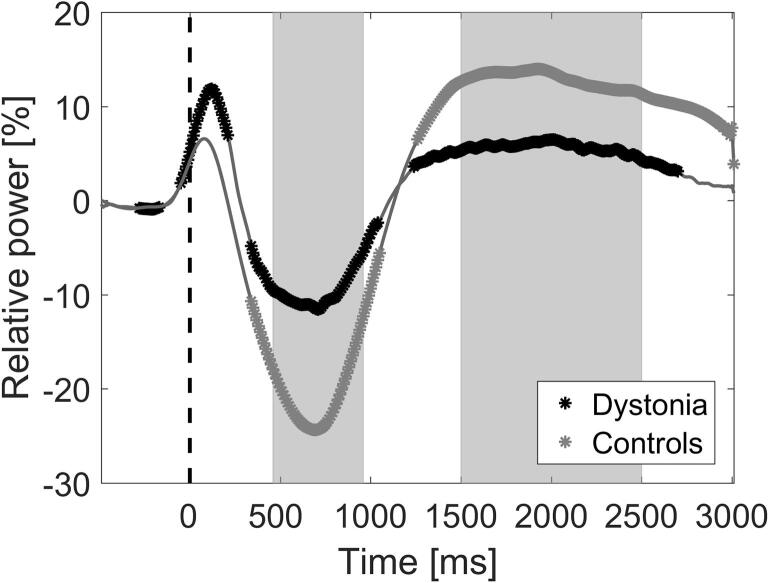
**Time course of the event-related changes in the alpha range over contralateral sensorimotor cortex**. Mean alpha (8–12 Hz) power at each time point as a percentage with respect to the baseline is shown separately for the dystonia and control groups. Points of significant departure from the baseline after false discovery rate correction for multiple comparisons are shown by black or grey* for dystonia and control groups respectively. The time windows for subsequent comparison between groups were derived to be centred around the peak ERD and ERS, as shown by shaded grey regions.

In order to compare event-related changes between groups, specific time windows for alpha ERD and alpha event-related synchronisation (ERS) were defined. This was done by plotting the time course of the mean event-related power in the alpha range, for both the control and dystonia groups, and identifying points of significant departure from the baseline, after false discovery rate correction for multiple comparisons (Fig. 4). The time windows were defined symmetrically around the average peak ERD and ERS across all subjects, giving windows of 0.46–0.96 s and 1.5–2.5 s post-stretch for ERD and ERS respectively. The mean levels of alpha ERD and alpha ERS were then calculated for each individual during these defined windows to allow comparison between groups. Although the focus of this paper is alpha/mu range activity, changes in beta band (14–30 Hz) activity were also calculated for completeness and are reported in the Supplementary material.

### 2.6 Statistics

Statistical analyses were performed in SPSS version 25. The data were assessed for normality (Shapiro-Wilk test) and non-parametric analyses were used where applicable.

Our key a-priori hypotheses were that levels of alpha ERD and ERS over sensorimotor cortex in response to the proprioceptive stimulus would differ between the three groups: controls, isolated genetic/idiopathic dystonia and dystonic CP. We tested these hypotheses separately for the dominant and non-dominant hands. Where cross-group comparisons were significant, post-hoc comparisons were made between pairs of groups. Statistical analyses were also performed to compare levels of alpha ERD and ERS across different age groups in typically developing children to assess the apparent developmental changes observed across this group. For all these analyses (including the post-hoc comparisons where applicable) the Benjamini-Hochberg procedure was applied with a false discovery rate of 5% to account for multiple comparisons (Benjamini and Hochberg, 1995) and the cut-off for statistical significance was adjusted accordingly.

Exploratory analyses were also performed to compare levels of alpha ERD and ERS across specific dystonia aetiological sub-groups and to investigate a potential relationship between spectral changes and measures of dystonia severity. Levels of resting power in each frequency band were also compared between groups. A significance level of p < 0.05 was accepted for these secondary analyses.

Comparisons between mean levels of alpha ERD or ERS across groups were made using Analysis of Covariance (ANCOVA), controlling for age and wrist excursion as covariates.

Differences between paired data (e.g. contralateral versus ipsilateral response) were assessed using paired t-tests. Correlation analyses were performed using Pearson or Spearman correlation as applicable. Sensitivity analyses to check for potential confounding factors between groups (age, wrist excursion, level of background EMG) were performed using a one-way ANOVA or Kruskal Wallis test.

## 3 Results

All controls (N = 22) performed the task successfully with both hands. 35 patients with dystonia initially attempted to participate in the study. One abandoned the study due to feeling unwell, whilst four patients struggled with the task due to excessive involuntary movements and the data were heavily contaminated by movement and muscle artefacts. Hence the data from these 5 participants were excluded. Of the remaining 30 patients, 5 were unable to complete the task satisfactorily with their non-dominant hand. Thus dominant hand data were available for 30 patients and non-dominant hand data were available for 25 patients. Only participants with technically satisfactory EEG data are reported. Demographic and clinical details of these patients are reported in Table 1.

The dystonia group includes 20 patients with isolated genetic or idiopathic dystonias and 10 patients with dystonic CP. Mean age was 12.67 years (95% CI 11.47–13.88) and did not differ significantly between control, isolated genetic/idiopathic dystonia and dystonic CP groups (see Suppl Table S1). Mean wrist excursion was 10.34° from neutral (95%CI 9.96–10.72) and did not differ significantly between groups for either hand (see Suppl Table S1). Wrist excursion did however show a small reduction with increasing age across the whole group, which was statistically significant for the dominant hand, and was therefore included as a covariate in later analyses (see Suppl Table S2).

The paradigm involved a passive wrist extension and participants were instructed to relax the forearm as much as possible. However, complete relaxation is difficult to achieve, especially in the presence of a movement disorder. Levels of background EMG were therefore recorded from the forearm flexors and extensors and compared between groups. Mean levels of rectified EMG activity were slightly higher in individuals with dystonia than in controls, as would be anticipated (see Suppl Table S3). Comparing across groups, this difference reached statistical significance for the flexor but not the extensor muscles (Suppl Table S3). However, there was no correlation between the level of rectified EMG and the magnitude of alpha ERD or alpha ERS in either the control or dystonia groups.

The passive wrist movement evoked a clear and consistent potential over the contralateral sensorimotor cortex, maximal at C3, CP1, CP5 for right hand movement and at C4, CP2, CP6 for left hand movement. The response comprised a series of 3 positive and 3 negative peaks as shown in Fig. 1D-E and is comparable to stretch evoked potentials (StretchEPs) published elsewhere (Campfens et al., 2015). The peak-to-peak amplitudes of the main StretchEP components (Suppl Table S4) did not differ significantly between controls and participants with genetic/idiopathic dystonia. In individuals with dystonic CP, some of the StretchEP components were of reduced amplitude compared with controls (Suppl Table S4).

### 3.1 Time frequency analysis

Event-related changes in spectral activity are shown for an individual control subject in Fig. 1F-I. A clear response is seen over the hemisphere contralateral to right hand movement (seen over C3) and left hand movement (seen over C4). Changes over the ipsilateral hemisphere are minimal. Fig. 2 shows the pooled event-related changes in spectral activity for controls. Data from movement of each hand are shown, demonstrating a clear response over the contralateral sensorimotor cortex for both the dominant and non-dominant hands. Fig. 3 shows the equivalent plots for individuals with dystonia. Note, for both figures, the sharp increase in power with respect to baseline at time zero, extending up to 40 Hz, and the brief, early increase in theta range power from 0 to 300 ms are likely to reflect movement artefact and a contribution from the stretchEP. These changes are not considered further in the analysis.

The first physiological change is seen over the contralateral hemisphere as a clear event-related desynchronisation (ERD) in the alpha/mu range with peak around 700 ms. This is followed by an alpha/mu event-related synchronisation (ERS) with peak around 2 s post-stimulus. ERS in the beta range is also seen, with a slightly earlier window, approximately 1–2 s. The changes over the ipsilateral hemisphere are minimal (Fig. 2D-F and J-L, Fig. 3 D-F and J-L) and of significantly lower amplitude than those over the contralateral hemisphere (Suppl Table S5). The subsequent analysis focuses on changes over the hemisphere contralateral to the stretch. Fig. 4 delineates the time course of the event-related changes in the alpha range over contralateral sensorimotor cortex for each group, identifying points of significant departure from the baseline after false discovery rate correction for multiple comparisons. The time windows for subsequent comparison between groups were derived to be centred around the peak ERD and ERS as shown.

Dystonia patients with and without implanted DBS showed very similar patterns of event-related spectral changes with no significant differences in alpha ERD or ERS (see Suppl Table S6). These groups were therefore combined for subsequent analysis.

### 3.2 Comparison between controls and dystonia

**Fig. 5:**
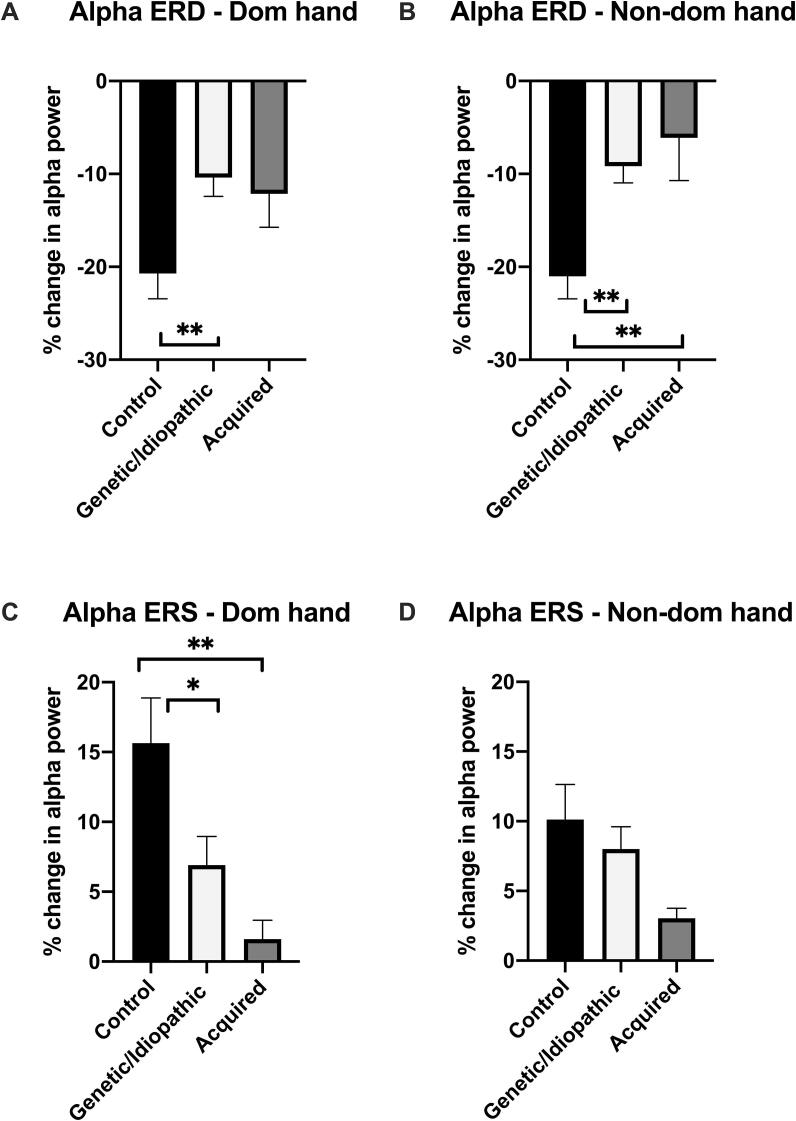
**Comparison of contralateral hemisphere alpha ERD and alpha ERS between controls (n = 22), genetic/idiopathic dystonia (n = 20) and acquired dystonia (n = 10)**. Bars show mean + SEM of % change in alpha power over contralateral sensorimotor cortex (C3/C4) contralateral to the stretch in the defined time windows (0.3–0.8 s post stimulus for ERD and 1.5–2.5 s post-stimulus for ERS). The difference in mean levels of alpha ERD across groups is significant for the non-dominant hand (ANCOVA controlling for age and excursion: F(2,42) = 6.388, p = 0.004). The difference in mean levels of alpha ERS across groups is significant for the dominant hand (ANCOVA controlling for age and excursion: F(2,48) = 7.829, p = 0.001). Bars and asterisks show results of post-hoc comparisons, where these were significant *p < 0.05 ** p < 0.01 See text for absolute values.

Mean levels of contralateral alpha ERD and ERS for control and dystonia groups are shown in Table 2 and Fig. 5. Levels of alpha ERD were significantly lower in dystonia compared with controls, with the difference being particularly striking for the non-dominant hand (ANCOVA across the 3 groups, controlling for age and wrist excursion: Non-dominant hand F(2,42) = 9.397, p = 0.000424; Dominant hand F(2,47) = 4.45, p = 0.017). Post-hoc comparisons showed that for the non-dominant hand the difference was significant for both genetic/idiopathic dystonia and dystonic CP: controls versus genetic/idiopathic dystonia: F(1,38) = 13.615, p = 0.001; controls versus dystonic CP: F(1,23) = 9.819, p = 0.005; genetic/idiopathic dystonia versus dystonic CP: F(1,21) = 0.728, p = 0.403). For the dominant hand the difference only reached significance for the genetic/idiopathic dystonia group: controls versus genetic/idiopathic dystonia: F(1,38) = 8.521, p = 0.006; controls versus dystonic CP: F(1,28) = 2.377, p = 0.134; genetic/idiopathic dystonia versus dystonic CP: F(1,26) = 0.187, p = 0.669).

**Table 2:** **Magnitude of alpha event related desynchronisation (ERD) and synchronisation (ERS) in controls, genetic/idiopathic and acquired dystonia/dystonic CP.** Mean levels of ERD and ERS for time windows 0.46–0.96 s and 1.5–2.5 s post-stimulus respectively, are shown for each group. All data relate to the hemisphere contralateral to the stretch. 95% confidence intervals are shown in brackets Results are presented for both dominant and non-dominant hand movement. (Note a small number of subjects showed only a very small and/or brief ERD or ERS response in the respective time window. In these subjects the mean level of relative power for ERD or ERS across the defined time window was therefore positive or negative respectively. As a consequence, the 95% confidence intervals straddle zero in some cases).

	Dominant hand			Non-dominant hand		
	Controls N = 22	Genetic/Idiopathic N = 20	Dystonic CP N = 10	Controls n = 22	Genetic/Idiopathic n = 20	Dystonic CP n = 5
Alpha ERD (%)	−20.72 (-26.38 to −15.05)	−10.39 (-14.61 to −6.16)	−12.13 (-20.29 to −3.98)	−21.01 (-26.04 to −15.97)	−9.16 (-12.94 to -5.37)	−6.11 (-18.90 to 6.69)
Alpha ERS (%)	15.64 (8.90 to 22.38)	6.91 (2.61 to 11.20)	1.61 (-1.43 to 4.66)	10.13 (4.91 to 15.35)	8.01 (4.65 to 11.36)	3.05 (1.09 to 5.01)

Mean levels of alpha ERS were also lower in dystonia compared with controls, with the lowest levels seen in the dystonic CP group (Table 2, Fig. 5C-D). For the dominant hand this difference was strongly significant: ANCOVA across the 3 groups, controlling for age and wrist excursion: F(2,47) = 7.786, p = 0.001. Post-hoc analyses: controls versus genetic/idiopathic dystonia: F(1,38) = 6.901, p = 0.012; controls versus dystonic CP: F(1,28) = 15.393, p = 0.001; genetic/idiopathic dystonia versus dystonic CP: F(1,26) = 2.847, p = 0.104. For the non-dominant hand, mean levels of alpha ERS were not significantly different between controls and either dystonia group, although there was a modest reduction in the dystonic CP group (ANCOVA across the 3 groups, controlling for age and wrist excursion: F(2,42) = 1.772, p = 0.182).

Beta ERD and ERS findings are presented in Suppl Table S7.

### 3.3 Controls – developmental profile

To assess the developmental profile of the control group, a comparison was made across three age groups: Group 1 (Young) (N = 10) age 5–9 years, Group 2 (Intermediate) (N = 6) age 10–14 years, Group 3 (Older) (N = 6) age 15–19 years. For both the dominant and non-dominant hand, the peak-to-peak amplitude of each of the main stretchEP components was generally slightly smaller in older children. However, this difference across age groups did not reach statistical significance for any of the components (Suppl Table S8).

The time–frequency plots show clear age-related changes in alpha/mu ERD and ERS in the control group (Fig. 2), which were minimal in the dystonia group (Fig. 3). In the 5–9 year age group (Fig. 2A and G), the alpha/mu ERD is the most prominent change over the contralateral hemisphere. In the 10–14 year old age group (Fig. 2B and H), this alpha/mu ERD is again present, and is followed by a strong alpha/mu ERS. Beta-range ERS is also seen in this age-group. In the 15–19 year old age group (Fig. 2C and I), the alpha/mu ERD and ERS are diminished relative to the 10–14 year olds, but the beta ERS appears more prominent.

**Fig. 6:**
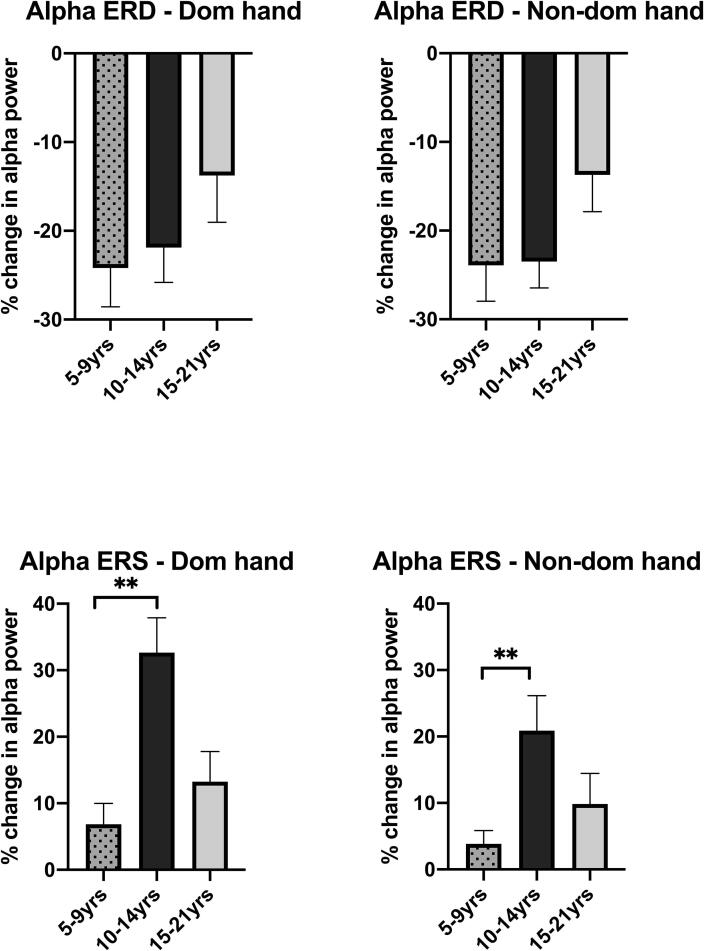
**Comparison of contralateral hemisphere alpha (8**–**12 Hz) ERD and alpha ERS across age-groups in typically developing children.** Data are shown for the Young age group (5–9 years, n = 10), Intermediate age group (10–14 years, n = 6) and Older age group (15–21 years, n = 6). Bars show mean + SEM of % change in alpha power over sensorimotor cortex (C3/C4) contralateral to the stretch in the defined time windows (0.3–0.8 s post stimulus for ERD and 1.5–2.5 s post-stimulus for ERS). There was a significant difference across age groups for alpha ERS in response to both dominant and non-dominant hand stretch (ANCOVA F(2,18) = 6.144, p = 0.009 and F(2,18) = 6.064, p = 0.010 respectively). Bars and asterisks show results of post-hoc comparisons between young and intermediate age groups ** p < 0.01.

Mean amplitudes of contralateral alpha ERD and ERS are shown for controls in each age group in Table 3 and Fig. 6. The amplitude of alpha ERD in the older age group was slightly lower than in the young and intermediate age groups for both hands, particularly the non-dominant hand (Fig. 6A-B). However, this difference was not statistically significant after controlling for multiple comparisons (ANCOVA across age group controlling for mean excursion: Dominant hand F(2,18) = 1.703, p = 0.210; Non-dominant hand F(2,18) = 3.629, p = 0.047).

**Table 3:** **Magnitude of alpha event related desynchronisation (ERD) and synchronisation (ERS) in controls, across age-groups.** Mean levels of ERD and ERS for time windows 0.46–0.96 s and 1.5–2.5 s post-stimulus respectively, are shown for controls in each age-group. All data relate to the hemisphere contralateral to the stretch. 95% confidence intervals are shown in brackets Results are presented for both dominant and non-dominant hand movement. (Note a small number of subjects showed only a very small and/or brief ERD or ERS response in the respective time window. In these subjects the mean level of relative power for ERD or ERS across the defined time window was therefore positive or negative respectively. As a consequence, the 95% confidence intervals straddle zero in some cases).

	Dominant hand			Non-dominant hand		
	Controls age 5–9 years	Controls age 10–14 years	Controls age 15–21 years	Controls age 5–9 years	Controls age 10–14 years	Controls age 15–21 years
Alpha ERD (%)	−24.18 (-34.07 to – 14.3)	−21.90 (-31.93 to −11.87)	−13.76 (-27.32 to −0.21)	–23.91 (–33.10 to −14.72)	–23.48 (-31.13 to-15.84)	−13.70 (-24.38 to 3.03)
Alpha ERS (%)	6.85 (-0.20–13.91)	32.65 (19.24 to 46.07)	13.26 (1.63 to 24.90)	3.86 (-0.62 to 8.34)	20.88 (7.35 to 34.41)	9.83 (-2.07 to 21.73)

In contrast, mean levels of alpha ERS showed a strong and significant increase between the young and intermediate age groups and then settled to an intermediate level in the older age group (Fig. 6 C-D). (ANCOVA across age group controlling for mean excursion: Dominant hand F(2,18) = 6.144, p = 0.009; Non-dominant hand F(2,18) = 6.064, p = 0.010). In post-hoc analyses, the increase between the young and intermediate age groups was statistically significant for both hands (ANCOVA controlling for excursion: Dominant hand F(1,13) = 9.697, p = 0.008; Non-dominant hand F(1,13) = 12.569, p = 0.004). The difference between the intermediate and older age groups was not significant after controlling for multiple comparisons (ANCOVA: Dominant hand F(1,9) = 5.830, p = 0.039; Non-dominant hand F(1,9) = 2.887, p = 0.124).

Peak frequency of alpha/mu ERD is shown in the Supplementary file (Suppl Fig S1) and beta ERD and ERS findings across control age-groups are presented in Suppl Table S9.

### 3.4 Analysis of aetiological dystonia sub-groups

**Fig. 7:**
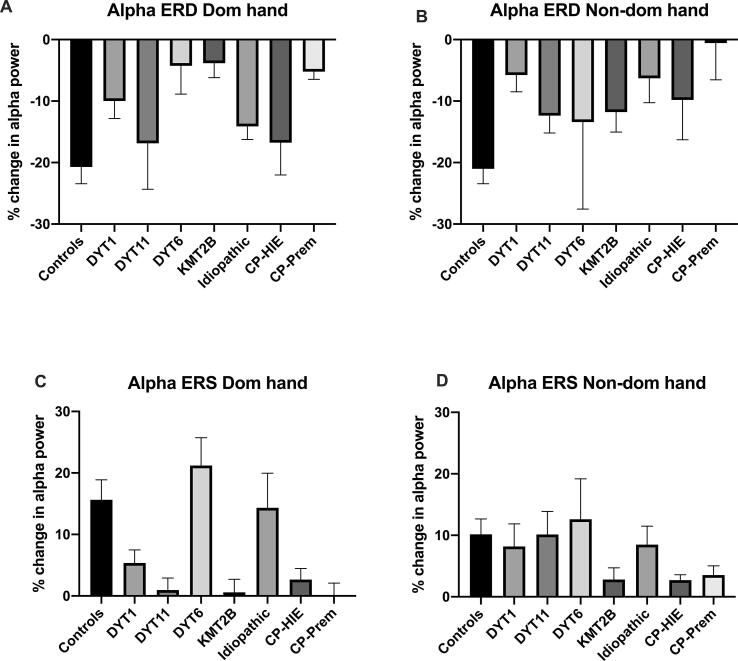
**Comparison of alpha ERD and ERS across aetiological sub-groups.** Comparison of alpha ERD (A and B) and alpha ERS (C and D) amplitudes between controls (n = 22) and aetiological sub-groups of dystonia: DYT1 (n = 6), DYT11 (n = 4), DYT6 (n = 2), KMT2B (n = 4), Idiopathic (n = 4), Dystonic Cerebral palsy due to term hypoxic ischaemic encephalopathy (CP-HIE) (n = 6 for dominant hand and n = 3 for non-dominant hand) and Dystonic Cerebral palsy due to prematurity (CP-Prem) (n = 4 for dominant hand and n = 2 for non-dominant hand). Bars show mean + SEM of % change in alpha power over contralateral sensorimotor cortex (C3/C4) in the defined time windows (0.3–0.8 s post stimulus for ERD and 1.5–2.5 s post-stimulus for ERS). Results of exploratory statistical analyses of sub-groups are reported in the supplementary information (Suppl Table S10).

As a secondary, exploratory analysis, the patterns of alpha ERD and ERS across different dystonia aetiologies were investigated. These findings are shown in Fig. 7A-B (alpha ERD) and Fig. 7C-D (alpha ERS). The results are presented in the supplementary information (Suppl Table S10) as preliminary findings. The small numbers mean that results will need to be confirmed in larger cohorts.

### 3.5 Resting power

In both the control and dystonia groups, resting levels of theta power, and to some extent alpha power, decreased with increasing age, while beta power increased with age (Suppl Table S11). In controls, a correlation was seen between the amplitude of alpha ERD and the resting level of alpha power, such that higher levels of resting alpha power are associated with stronger alpha ERD. Similarly, higher levels of resting alpha power are associated with stronger alpha ERS (Suppl Table S12). This relationship was not seen consistently in the dystonia groups (see Suppl Table S12). Further comparisons of resting power between controls and dystonia, pre- and post-DBS are presented in the Supplementary file (Suppl Table S13).

### 3.6 Relationship between spectral measures and severity of dystonia

BFMDRS-m scores and AMPS scores were available in 26 and 18 patients respectively.

Correlation analysis revealed no consistent relationship between magnitude of alpha ERD or alpha ERS and BFMDRS-m or AMPS (motor and process) scores for either the dominant or non-dominant hand. However, there was a significant positive correlation between BFMDRS-m scores and resting theta power (ie higher levels of power were associated with greater severity) for the genetic/idiopathic dystonia group (Rho = 0.518, p = 0.028) but not for the dystonic CP group (R = -0.2, p = 0.635).

## 4 Discussion

The present study explored cortical sensory processing in young people with dystonia, using a paradigm that focusses on changes in oscillatory neuronal activity, particularly within the alpha/mu band. The key and novel findings are:

1. Event-related changes in spectral activity over the sensorimotor cortex evoked by the proprioceptive stimulus show a series of developmental changes in typically developing children, with 5–9 year olds already showing prominent ERD in the alpha/mu range but the alpha ERS developing later, from 10 years onwards.
2. Young people with dystonia have a significantly reduced alpha ERD response compared with controls, particularly for the non-dominant hand.
3. Young people with dystonia show a greatly reduced alpha ERS response compared with controls, particularly in the dominant hand, and do not exhibit the clear developmental profile seen in typically developing children.
4. Patients with dystonic CP exhibit similar reductions in alpha ERD and ERS to those with isolated genetic/idiopathic dystonia, demonstrating that this is broadly a common feature in the pathophysiology of dystonia.

### 4.1 Event-related changes in alpha/mu activity and their development in typically developing children

The suppression of alpha range (8–12 Hz) activity over the sensorimotor cortex (usually referred to as mu activity in this topographical region) in relation to movement or somatosensory stimulation is well documented (Pineda, 2005, Demas et al., 2019) and there is an extensive literature on the development of the mu (or “central”) rhythm in infancy and childhood (see Demas et al for a recent comprehensive review^27^). The event-related suppression or desynchronisation is considered to reflect an excitation or activation of the relevant cortical area or network (Pineda, 2005, Pfurtscheller and Lopes da Silva, 1999, Neuper and Pfurtscheller, 2001), triggered by movement preparation or processing of sensory information. The ERD is usually followed by a rebound in mu activity (ERS), which in the past was thought to reflect an “idling” rhythm, but is more recently considered to reflect active inhibition of the motor network or a resetting of the sensorimotor system ready for subsequent activities (Pineda, 2005, Demas et al., 2019, Gaetz et al., 2010, Gaetz et al., 2011). Modulation of mu activity also occurs during *observation* of a movement and is therefore considered to reflect activity within the human mirror neuron system (Cannon et al., 2014; Fox et al., 2016, Braadbaart et al., 2013; Lepage and Theoret, 2006; Woodruff and Klein, 2013). However, the degree of mu suppression is greater when the observed action is goal-orientated (Lepage and Theoret, 2006) or when observing a movement performed live rather than on video (Ruysschaert et al., 2013), and *active* movement leads to greater mu desynchronisation than *observed* motor activity (Cannon et al., 2014).

The event-related alpha/mu changes in the current study are concordant with the above observations as they were temporally related to the passive wrist movement and were seen specifically over the contralateral sensorimotor cortex. A developmental equivalent to the mu rhythm has been demonstrated in young children and even in infants, with 6–9 Hz sensorimotor cortex activity displaying typical characteristics of adult mu in relation to movement and somatosensory stimulation, emerging around the end of the first year of life (Marshall et al., 2002, Thorpe et al., 2016, Berchicci et al., 2011). In infants, the findings are based on spontaneous mu activity, but several studies demonstrate characteristic event-related modulation of mu activity in pre-school aged children (as young as 2 years old) performing standardised motor tasks (Berchicci et al., 2011, Cheyne et al., 2014, Johnson et al., 2019). The peak frequency of the mu rhythm shows a rapid increase with age during the first year of life, and then continues to increase, more slowly during pre-school and early school-aged children up to adolescence and adulthood (Thorpe et al., 2016, Berchicci et al., 2011). We observed a similar trend of increasing peak frequency of mu ERD with increasing age (Suppl Fig. S1). The mu ERD and ERS in our study also showed a correlation with resting levels of alpha/mu activity, even when controlling for age (Suppl Table S12), i.e. the higher the level of resting alpha/mu power, the stronger the alpha/mu modulation seen, both for ERD and ERS. Trevarrow et al. report a similar association for beta ERD (Trevarrow et al., 2019), in an active movement task.

Most studies reporting modulation of the mu rhythm and its development employ an active motor task, and show that mu suppression tends to occur in advance of the movement onset (Gaetz et al., 2010, Cheyne et al., 2014, Trevarrow et al., 2019). In contrast, the current study used a passive, rather than an active movement, and the corresponding alpha/mu ERD is seen *after* the movement (Fig. 2, Fig. 3, Fig. 4). Similar temporal changes are seen in studies of mu suppression following somatosensory input in the form of electrical stimulation of the median nerve (Pihko et al., 2014). However, the current paradigm uses passive movement/stretch as the stimulus and therefore targets more specifically the proprioceptive sensory fibres. The development of proprioception in children has not yet been fully explored (Holst-Wolf et al., 2016), so the control group findings in this study, which demonstrate a clear developmental profile in spectral activity over the sensorimotor cortex following this particular stimulus, add to the literature in this field, as well as providing an important back-drop against which to study spectral measures of sensory/proprioceptive processing in dystonia, many forms of which have childhood onset, and in which abnormalities of proprioceptive sensory processing are considered of particular importance (Kaji et al., 1995).

Our results indicate that the alpha/mu ERD in response to the stretch stimulus develops at an earlier age (seen in the 6–9 year old age group) than the subsequent alpha/mu ERS, which was seen in children from 10 years upwards (Fig. 2, Fig. 6). These findings agree with a MEG study of mu development measured in response to a button press task, which also found that mu/alpha ERD appears more prominently than mu/alpha ERS in young children (3–5 years), whereas a clear alpha/mu ERS has developed by adulthood (Johnson et al., 2019). Importantly, alpha/mu ERS in the current study peaked in amplitude in the intermediate 10–14 year old (pubertal) age group and then reduced again in the older (15–21 year old) age group, although still at higher levels than in the youngest children (Fig. 2, Fig. 6). It is possible that these findings reflect immaturity of inhibitory mechanisms, which become more refined towards later adolescence. It is well-established that levels of motor cortical inhibition are significantly lower in children than adults (Mall et al., 2004, Garvey and Mall, 2008) and that sensorimotor development continues throughout childhood and well into adolescence (Quatman-Yates et al., 2012). Gehringer et al. (Gehringer et al., 2019) demonstrated that adolescents show a more enhanced modulation of alpha–beta cortical oscillations in response to a somatosensory stimulus applied to the lower limb during isometric force production compared with adults (Gehringer et al., 2019). Although our results are not directly comparable with that study since the paradigm and the temporal pattern of activity were different, this provides another example of immature processing of sensorimotor cortical oscillations in adolescence.

### 4.2 Event-related changes in beta range activity

The current study focuses on event-related changes in alpha/mu activity since cortical and sub-cortical oscillations in the alpha and theta range have been shown to be abnormal in dystonia. However, the stretch stimulus also evoked clear sensorimotor cortex EEG changes in the beta range, comprising a beta ERD, shorter-lived than the alpha ERD, and a beta ERS which was both earlier and shorter-lived than the alpha ERS (see Fig. 2). Event-related beta ERD and ERS in relation to movement are well-described phenomena (Demas et al., 2019, Trevarrow et al., 2019). In the current study the changes relate to a passive movement, and the relative time courses of alpha and beta modulation are similar to those described by Pihko et al in relation to electrical stimulation of the median nerve (Pihko et al., 2014). Developmental studies have demonstrated an increase in amplitude of movement-induced beta ERD and ERS with age from pre-school to school-age children and a further increase in amplitude in adults (Gaetz et al., 2010, Trevarrow et al., 2019). The current study shows a similar developmental profile for beta ERD and ERS in response to the proprioceptive stimulus, with the strongest beta ERD and ERS occurring in the oldest age group (Fig. 2C & I).

As with alpha/mu ERD, beta ERD is considered to reflect activation of the sensorimotor networks (Pineda, 2005, Pfurtscheller and Lopes da Silva, 1999) while beta ERS is thought to reflect active inhibition within the sensori-motor cortex after termination of movement (Trevarrow et al., 2019), or more specifically the erasing of working memory information at the end of a task (Schmidt et al., 2019). Another possibility is that the strong post-movement beta ERS in sensorimotor cortex reflects the integration of feedback and reward (Schmidt et al., 2019). The event-related changes in these two frequency ranges appear to have different topographies. Whilst the current study used limited scalp EEG and is unable to more precisely localise the alpha and beta changes seen over the sensorimotor cortex, previously reported MEG studies using beamforming methodologies have demonstrated that alpha (alpha-mu) ERD is localised to the post-central gyrus (primary somatosensory cortex) while beta ERD and ERS are localised more anteriorly in pre-central primary motor cortex (Trevarrow et al., 2019).

### 4.3 Mu alpha ERD and ERS responses are diminished in young people with dystonia

An important and novel finding from the current study is that the modulation of alpha/mu activity over contralateral sensorimotor cortex in response to a proprioceptive stimulus is reduced in young people with dystonia compared with typically developing children (Fig. 5, Fig. 6). This was seen across all age groups, most prominently for ERD in response to non-dominant hand stretch (which was usually the more affected hand in the presence of a clinical asymmetry). The reduction in ERS was seen more prominently in response to dominant hand stretch (Fig. 5), where the reduction is particularly striking for the acquired dystonia/dystonic CP group.

Abnormalities of alpha/mu modulation have been described in individuals with hemiplegic cerebral palsy (Pihko et al., 2014, Inuggi et al., 2018, Kurz et al., 2014), but the literature in dystonia is sparse. In dystonia, several studies have shown impaired cortical *beta* desynchronisation during a movement task (Toro et al., 2000, Crowell et al., 2012, Kristeva et al., 2005). Kristeva et al. (Kristeva et al., 2005) suggested this abnormal activation of the beta range cortical oscillatory network may reflect impaired modulation of intra-cortical inhibition (Kristeva et al., 2005). However, no significant differences in alpha range spectral activity were observed in those studies. In contrast, Kukke et al. (Kukke et al., 2015) found reduced alpha-range ERD during an isometric extension of the more affected wrist in eight patients with hemidystonia following childhood stroke, and this reduction in ERD correlated with reduced force production (Kukke et al., 2015). The finding of an abnormality in the alpha rather than beta range was postulated to reflect the ongoing requirement for afferent feedback during a sustained isometric contraction, with alpha/mu modulation reflecting sensory feedback (Kukke et al., 2015).

The paradigm used in the current study involved a passive movement, allowing us to focus on spectral changes related to sensory processing, and demonstrated clear abnormalities in event-related alpha/mu modulation in dystonia. There is strong evidence that sensory processing is abnormal in dystonia (Tinazzi et al., 2000, Tinazzi et al., 2003, Avanzino et al., 2015, Hallett, 1995, Kaji et al., 2004), but this has rarely been studied in the context of modulation of cortical oscillatory activity. Given the growing evidence of pathologically enhanced cortical and sub-cortical oscillations in the alpha-theta range in dystonia (Neumann et al., 2012, Neumann et al., 2015, Sharott et al., 2008, Barow et al., 2014, Miocinovic et al., 2017), the relevance of alpha/mu oscillatory activity in sensory processing (Pineda, 2005, Demas et al., 2019, Trevarrow et al., 2019), and evidence that proprioceptive feedback is abnormal in dystonia (Kaji et al., 1995), we were keen to investigate alpha/mu modulation in response to a proprioceptive task in our patients.

In previous work we identified abnormalities of standard clinical SEPs in up to 47% of young people with dystonia, predominantly those with acquired dystonias (McClelland et al., 2018). We interpreted this in the context of the network model of dystonia: whilst individuals with genetic/idiopathic dystonias show abnormal gating of sensory inputs (Tinazzi et al., 2000, Frasson et al., 2001), many children with dystonia arising from perinatally acquired brain injury (i.e. dystonic cerebral palsy) have abnormalities earlier still in the pathway, with even the arrival of afferent information at the sensory cortex being disrupted (McClelland et al., 2018). However, standard SEPs were still normal in approximately 53% of children with dystonia, hence the need to investigate measures of sensory processing in these individuals. In the current study, most participants with dystonia had normal standard clinical SEPs (76% of those tested) and none had absent SEPs, probably due to the nature of the experimental protocol, which could not be undertaken by more severely affected children. This is reflected by the observation that the amplitude of the stretch EP was not significantly different between controls and the dystonia groups, although there was a trend towards lower amplitudes in dystonic CP (Suppl Table S4). Despite the stretch EP being largely consistent across subjects, indicating effective stimulation, a clear reduction in alpha ERD was seen in individuals with dystonia, suggesting impaired processing of the proprioceptive information relating to the stretch stimulus.

Reduced alpha ERD in response to a somatosensory stimulus has been associated with age-related decline in sensory gating in healthy aging (Cheng et al., 2015). It is therefore possible that the reduced alpha ERD observed in the current study could contribute to impaired sensory gating in dystonia. This phenomenon has been studied comprehensively in adults with primary and focal dystonias using paired pulse paradigms (Tinazzi et al., 2000, Frasson et al., 2001).

Children with dystonia also showed impaired alpha ERS following the stimulus (Fig. 5, Fig. 6). One could argue that if the system has not desynchronised then there is no need to re-synchronise. However, the ERS in controls is not just a passive return to baseline, but shows a clear increase in alpha power above baseline levels, and is thought to involve active inhibitory mechanisms to reset the sensorimotor network following stimulation (Pineda, 2005, Demas et al., 2019, Gaetz et al., 2010, Gaetz et al., 2011). The current findings therefore suggest that those cortical inhibitory processes involved in resetting the network are disrupted in young people with dystonia following a proprioceptive stimulus.

### 4.4 Differences and similarities in neurophysiological features between isolated genetic/idiopathic dystonias and dystonic CP

A further important finding in this study is that the abnormal modulation of alpha/mu activity was seen in both isolated genetic/idiopathic and dystonic CP. There is a clear discrepancy between these groups in responsiveness to neuromodulation with pallidal DBS, which likely reflects differences in underlying pathophysiology (Neychev et al., 2011). Despite this, most neurophysiological studies focus on isolated genetic/idiopathic dystonias and relatively little is known about the underlying pathophysiology of dystonic CP. A few studies have addressed this question and have demonstrated differences between genetic and acquired dystonias in measures of corticospinal excitability, cortical inhibition and plasticity (Kojovic et al., 2013, Trompetto et al., 2012), patterns of globus pallidus neuronal firing (McClelland et al., 2016) and patterns of beta-range corticomuscular coherence and its modulation (McClelland et al., 2020). In contrast, elevated low-frequency (4–12 Hz) intermuscular coherence is a common feature across different dystonia aetiologies (McClelland et al., 2020, Grosse et al., 2004).

A better understanding of the differences and common pathophysiological features between different types of dystonia could help explain differences in responsiveness to therapy and is needed in order to develop a more individualised approach to neuromodulation and other therapeutic interventions. The current findings contribute to this goal by demonstrating an abnormality of spectral oscillatory activity relating to sensory processing which is common between isolated genetic/idiopathic dystonias and dystonic CP. The preliminary analysis across aetiological sub-groups further suggests that impaired alpha/mu modulation by proprioceptive stimuli is common to several specific genetic or acquired aetiologies (Fig. 7 and Suppl Table S10). However, the numbers in these sub-groups are small and further studies are warranted to explore these possible differences in more detail.

### 4.5 DBS appears to correct the pathologically enhanced levels of theta oscillations but not the abnormal mu modulation.

Resting levels of EEG power in the theta range were elevated in pre-DBS dystonia patients compared with controls. In contrast, the post-DBS group had significantly lower levels of theta power than the pre-DBS group (Suppl Table S13). These findings are consistent with other reports showing pathologically enhanced low-frequency (4–12 Hz) cortical and sub-cortical oscillations in dystonia (Sharott et al., 2008, Neumann et al., 2015), which are reduced by DBS (Barow et al., 2014, Miocinovic et al., 2017), and extends these findings to children with both isolated genetic/idiopathic dystonia and dystonic CP.

Although resting levels of cortical oscillations were improved by DBS, we did not detect any difference in the levels of alpha/mu modulation in relation to the proprioceptive stimulus between the pre-DBS and post-DBS groups (Suppl Table S13). The reasons for this are unclear. It is noted that we compared separate groups of pre-DBS and post-DBS patients, and it is possible that small intra-individual differences would be detected in a larger group of patients with paired pre- and post-DBS data.

The appearance of mu rhythm over sensorimotor cortex during early life appears to relate to the development of motor skills, with the relative amplitude of the central 6–9 Hz rhythm peaking around 2 years of age, during a period of intense locomotor development (Marshall et al., 2002). Another study found a correlation between infant mu and crawling onset (Xiao et al., 2017). Interestingly, Strogonova et al. (Stroganova et al., 1999) show evidence that the central 6–9 Hz rhythm in infants is more strongly correlated with corrected gestational age, whereas posterior alpha is more strongly correlated with chronological age. The authors speculate that this could be explained by development of the alpha rhythm being influenced by visual stimuli, which the infant is exposed to only after birth, whereas mu rhythm development is influenced by somatosensory/sensorimotor stimulation, which is experienced even within the uterus (Stroganova et al., 1999). The observation that within our cohort the small group with dystonic CP due to prematurity show lower levels of mu/alpha modulation than most other aetiological groups (Fig. 7), could be seen as concordant with this hypothesis.

It is interesting that we observed similar patterns of abnormality in individuals with genetic dystonia with onset in mid-childhood, and in those with perinatal onset dystonia. The abnormality was just as striking in those who had recently been diagnosed (<1year) with DYT1 as in those who had a perinatal onset lesion, and so did not relate to duration of dystonia. Although the typical onset of DYT1 dystonia is in mid-childhood, development of normal sensorimotor circuitry may already have been disrupted. This is supported by evidence from murine studies demonstrating that deletion of Torsin A leads to selective neurodegeneration in sensorimotor circuits in early post-natal life (Liang et al., 2014).

We did not detect any direct correlation between levels of mu/alpha modulation and dystonia severity, measured using the BFMDRS-m, in this study. In contrast, studies in children with hemiplegic CP have found a clear relationship between aberrant synchronisation of alpha/mu activity in sensorimotor cortex and motor performance (Kurz et al., 2014, Kurz et al., 2015). Further studies in children with dystonia should therefore investigate levels of alpha/mu modulation in relation to performance of more goal-orientated tasks.

### 4.6 Study limitations

Although the Hi5 device was designed to produce a consistent excursion across the age-range studied, a small but significant decrease in excursion with age was noted. Several factors may have contributed, including increased weight and therefore inertia of the hand in older children, and reduced joint compliance with increasing age (Lin et al., 1994, Lin et al., 1996). Excursion was therefore taken into account as a covariate in the analysis. Importantly, there was no significant difference in excursion between controls and individuals with dystonia.

Ideally, we would have pre-DBS and post-DBS data from the same subjects. However, owing to the relatively small numbers of children going forward for DBS, achieving an adequate sample size for this type of study takes many years and this is on-going work. The possibility that ongoing DBS degraded the pattern of the EEG response to stretch should also be acknowledged, and we did not use advanced methods of artefact removal in this study (Lio et al., 2018). Nevertheless, spectral power at rest tended to be lower in amplitude in the theta and alpha bands in those patients with DBS compared with those without DBS, and there was no difference in the beta band. In addition, the magnitude of alpha/mu ERD and ERS in individuals with dystonia with and without DBS did not differ. These observations would suggest that EEG contamination at the frequencies of interest in the current study was relatively limited.

The clinical group is heterogeneous in terms of aetiology. Preliminary analyses suggest possible differences in patterns of mu modulation across some of the aetiological sub-groups, but this will require on-going investigation with larger sample sizes. However, the sample reflects the clinical population and allows us to demonstrate physiological abnormalities that are common across different sub-types of dystonia, an important factor when considering possible biomarkers for monitoring of neuromodulation.

### 4.7 Study strengths

Whilst most other studies investigate cortical oscillations during rest or an ongoing movement task, this study takes a novel approach and explores dynamic, event-related changes in cortical oscillations. The paradigm also allowed us to link two important aspects of dystonia pathophysiology, i.e. abnormal cortical processing of sensory information and abnormal patterns of oscillatory activity. By choosing a passive movement task, we were able to focus on cortical processing of proprioceptive information and to minimise the potential confounding effects of motor command, planning and strategy, which may well be different in individuals with dystonia due to compensatory mechanisms.

## 5 Conclusion

Paediatric dystonias are an under-researched field but represent an important clinical need, especially with regards to acquired dystonias and dystonic CP, which begin in childhood but persist throughout the life-span. Further, these individuals offer an opportunity to better understand dystonias, which often begin in childhood, in the context of the developing brain. This study provides new pathophysiological insights, providing evidence for abnormal modulation of cortical alpha/mu activity in response to a passive movement, both in isolated genetic/idiopathic and acquired dystonias/dystonic CP. Given the importance of mu modulation in motor learning, including its proposed role within the mirror neuron system, these findings are likely to be relevant for understanding some of the impairments of motor control encountered in dystonia. Finally, the observation that abnormal alpha/mu modulation is common across different sub-types of dystonia, could provide a lead for future biomarker investigation and warrants further investigation.

## Funding

This work was supported by the Medical Research Council (MR/P006868/1 and MC_UU_12024/1), the Rosetrees Trust (A1598) and in part by the EU H2020 ICT-871767 REHYB project.

## CRediT authorship contribution statement

**Verity M McClelland:** Methodology, Investigation, Validation, Formal analysis, Writing - original draft, Project administration, Funding acquisition. **Petra Fischer:** Methodology, Software, Formal analysis. **Eleonora Foddai:** Investigation, Project administration. **Sofia Dall’Orso:** . **Etienne Burdet:** . **Peter Brown:** Formal analysis, Supervision. **Jean-Pierre Lin:** Resources, Investigation, Supervision.

## Declaration of Competing Interest

Jean-Pierre Lin has received educational support and consultancy fees from Medtronic Ltd.

Peter Brown has also received consultancy fees from Medtronic Ltd.
