# Supplementary material for: EEG measures of sensorimotor processing and their development are abnormal in children with isolated dystonia and dystonic cerebral palsy
Source: Neuroimage Clin. 2021 Jan 19;30:102569. doi: 10.1016/j.nicl.2021.102569 (PMC8044718; doi:10.1016/j.nicl.2021.102569)
Supplement: Supplementary data 1 [file mmc1.docx]

**Supplementary Data File**

**Section 1. Sensitivity analyses – Age, wrist excursion and background EMG data across groups.**

**Table S1. Age and wrist excursion comparison across groups**

|  | **Controls**  **N=22** | **Genetic/Idiopathic**  **N=20** | **Acquired**  **N=11** | **ANOVA** |
| --- | --- | --- | --- | --- |
| **Age (years)**  **Mean**  **and 95% confidence intervals** | 11.55  (9.69 – 13.41) | 13.16  (11.20-15.12) | 14.16  (10.84-17.49) | F=1.492  P=0.235 |
| **Wrist excursion (degrees)**  **Dominant hand.**  **Mean and 95% confidence intervals** | 10.85  (10.25-11.46) | 10.37  (9.54-11.21), | 9.88  (8.93-10.83) | F=1.448 p=0.245 |
| **Wrist excursion (degrees)**  **Non-dominant hand.**  **Mean and 95% confidence intervals** | 10.43  (9.91-10.97) | 10.25  (9.47-11.03) | 9.41  (6.66-12.16) | F=0.931 p=0.402 |

**Table S2. Wrist excursion comparison across age-groups (whole cohort)**

|  | **5-9 year olds**  **N=17** | **10-14 year olds**  **N=17** | **15-21 year olds**  **N=18** | **ANOVA** |
| --- | --- | --- | --- | --- |
| **Wrist excursion (degrees)**  **Dominant hand.**  **Mean and 95% confidence intervals** | 11.46  (10.98-11.95) | 10.14  (9.26-11.03) | 9.88  (9.15-10.60) | F=6.236  p=0.004 |
| **Wrist excursion (degrees)**  **Non-dominant hand.**  **Mean and 95% confidence intervals** | 10.73  (10.18-11.29) | 10.31  (9.47-11.16) | 9.64  (8.63-10.64) | F 2.201  p=0.123 |

**Table S3. Background levels of mean rectified EMG (µV) – Median values presented as data non-normally distributed**

|  | **Median level of rectified EMG (µV)** | | |  |
| --- | --- | --- | --- | --- |
|  | **Controls**  **N=22** | **Genetic/Idiopathic**  **N=20** | **Acquired**  **N=11** | **Kruskal Wallis test** |
| **Extensor Dominant hand** | 5.25  IQR 6.39 | 8.24  IQR 13.12 | 8.40  IQR 22.80 | H=1.888 p=0.389 |
| **Flexor Dominant hand** | 4.58  IQR 4.60 | 8.86  IQR 12.57 | 6.71  IQR 14.57 | H=7.717 p=0.021 |
| **Extensor**  **Non-Dominant hand** | 4.95  IQR 1.98 | 8.21  IQR 13.49 | 7.67  IQR 13.14 | H=5.143 p=0.07 |
| **Flexor**  **Non-Dominant hand** | 5.12  IQR 2.73 | 9.56  IQR 24.5 | 9.94  IQR 16.85 | H=6.853 p=0.032 |

**Section 2. Comparisons between Controls, genetic/idiopathic dystonia and acquired dystonia/dystonic CP.**

**Table S4.** **Stretch Evoked Potential - mean peak amplitudes (µV) (95% confidence limits)**

|  | **Dominant hand** | | | | **Non-dominant hand** | | | |
| --- | --- | --- | --- | --- | --- | --- | --- | --- |
|  | **Controls**  **n=22** | **Genetic/**  **Idiopathic**  **n=20** | **Dystonic CP n=10** | **ANCOVA controlling for age and excursion** | **Controls**  **n=22** | **Genetic/**  **Idiopathic**  **n=20** | **Dystonic CP**  **n=5** | **ANCOVA controlling for age and excursion** |
| **P1** | 4.84  (3.85 – 5.83) | 5.37  (4.14 – 6.59) | 3.00  (1.29 – 4.73) | F=3.199  p=0.050 | 6.03uV  (4.43-7.63) | 5.53  (4.19 – 6.87) | 3.06  (-1.30-7.42) | F=1.449  p=0.246 |
| **N1** | -5.19  (-6.42 - -3.96) | -5.07  (-6.65- -3.50) | -4.85  (-6.47- -3.24) | F=0.080  p=0.923 | -6.07  (-7.75 - -4.39) | -5.81  (-7.69 - -3.92) | -4.04  (-6.97 - -1.12) | F=0.377  p=0.688 |
| **P2** | 6.09  (3.64 – 8.53) | 5.85  (3.56 – 8.14) | 4.48  (2.57-6.38) | F=0.144  p=0.866 | 6.65  (3.29 – 10.01) | 6.91  (4.42 – 9.40) | 4.57  (1.17-7.96) | F=0.446  p=0.643 |
| **N2** | -13.96  (-17.60--10.31) | -12.83  (-16.89 – 8.78) | -8.27  (-12.41--4.13) | F=0.780  p=0.464 | -15.43  (-19.87--10.98) | -13.10  (-16.99- -9.20) | -6.17  (-10.74--1.60) | F=1.937  p=0.157 |
| **P3** | 14.54  (11.09 -18.00) | 10.95  (7.91 – 13.99) | 8.94  (4.57 – 13.30) | F=1.294  p=0.284 | 15.12  (11.26 -18.97) | 11.03  (7.66 – 14.41) | 4.99  (1.85 – 8.13) | F=3.384  p=0.043 |
| **N3** | -10.44  (-12.56- -8.33) | -7.88  (-10.32 - -5.44) | -6.64  (-9.56 - -3.72) | F=1.236  p=0.300 | -11.33  (-14.00 --8.65) | -9.68  (-12.48 - -6.88) | -5.39  (-8.32- -2.46) | F=2.562  p=0.089 |

**Table S5. Spectral changes over ipsilateral hemisphere – Control Group**

|  | **Contralateral**  **hemisphere** | **Ipsilateral hemisphere** | **Paired t-test** |
| --- | --- | --- | --- |
| **Alpha ERD (%)**  **Dominant hand**  Mean and 95% confidence limits | -20.72  (-26.38 to 15.05) | -10.05  (-15.56 to -4.54) | t=-3.4  p=0.002 |
| **Alpha ERD (%)**  **Non-dominant hand**  Mean and 95% confidence limits | --21.01  (-26.04 to 15.97) | -6.87  (-13.33 to 0.40) | t=-3.6  p=0.002 |
| **Alpha ERS (%)**  **Dominant hand**  Mean and 95% confidence limits | 15.64  (8.90 to 22.38) | -0.10  (-2.92 to 2.71) | t=4.268  p=0.000342 |
| **Alpha ERS (%)**  **Non-dominant hand**  Mean and 95% confidence limits | 10.13  (4.91 to 15.35) | 1.48  (-0.95 to 3.91) | t=3.309  p=0.003 |

**Table S6. Magnitude of alpha event related desynchronisation and synchronisation in individuals with dystonia with and without DBS.** Mean levels of alpha ERD and ERS (for time windows 0.46-0.96s and 1.5-2.5s post-stimulus respectively) are shown for each group. All data relate to the hemisphere contralateral to the stretch. 95% confidence intervals are shown in brackets Results are presented for both dominant and non-dominant hand movement.

|  | **Dominant hand** | | | **Non-dominant hand** | | |
| --- | --- | --- | --- | --- | --- | --- |
|  | **Pre-DBS**  **N=11** | **Post-DBS**  **N=19** | **ANCOVA** | **Pre-DBS**  **N=11** | **Post-DBS**  **N=14** | **ANCOVA** |
| **Alpha ERD (%)** | -13.57  (-22.14 to -5.00) | -9.46  (-13.05 to -5.88) | F=0.549  p=0.465 | -7.91  (-12.71 to -3.11) | -9.04  (-14.56 to -3.54) | F=0.986  p=0.332 |
| **Alpha ERS (%)** | 4.01  (0.22 to 7.80) | 5.80  (1.24 to 10.36) | F=0.119 p=0.732 | 5.72  (3.02 to 8.42) | 8.03  (3.25 to 12.82) | F=0.378  p=0.545 |

**Table S7. Magnitude of beta event related desynchronisation (ERD) and synchronisation (ERS) in controls, genetic/idiopathic and acquired dystonia/dystonic CP.** Mean levels of ERD and ERS for time windows 0.3-0.8s and 1.0-2.0s post-stimulus respectively, are shown for each group. 95% confidence intervals are shown in brackets . All data relate to the hemisphere contralateral to the stretch. Results are presented for both dominant and non-dominant hand movement.

|  | **Dominant hand** | | | | **Non-dominant hand** | | | |
| --- | --- | --- | --- | --- | --- | --- | --- | --- |
|  | **Controls**  **n=22** | **Genetic/**  **Idiopathic**  **n=20** | **Dystonic CP**  **n=10** | **ANCOVA** | **Controls**  **n=22** | **Genetic/**  **Idiopathic**  **n=20** | **Dystonic CP**  **n=5** | **ANCOVA** |
| **Beta ERD (%)** | -5.14  (-6.94 to -3.34) | -4.44  (-7.23- to -1.65) | -4.85  (-7.45 to -2.25) | F=0.082  p=0.921 | -4.06  (-6.23 to 1.89) | -4.30  (-6.08 to 2.52) | -2.29  (-4.17 to 0.40) | F=0.870  p=0.426 |
| **Beta ERS (%)** | 7.00  (4.25 to 9.75) | 5.24  (3.08 to 7.41) | 3.03  (-1.38 to 7.44) | F=3.968  p=0.026 | 7.75  (4.34 to 11.15) | 5.07  (2.38 to 7.77) | 2.27  (-4.99 to 9.53) | F=2.594  p=0.087 |

**Section 3. Comparison of findings across age-groups in Controls**

**Table S8.** **Stretch Evoked Potential – mean peak amplitudes (µV) in controls, by age-group**

|  | **Dominant hand** | | | | **Non-dominant hand** | | | |
| --- | --- | --- | --- | --- | --- | --- | --- | --- |
|  | Controls  5-9 years  n=10 | Controls  10-14 years  n=6 | Controls  15-21 years  n=6 | ANCOVA controlling for excursion | Controls  5-9 years  n=10 | Controls  10-14 years  n=6 | Controls  15-21 years  n=6 | ANCOVA controlling for excursion |
| **P1** | 5.09 | 5.84 | 3.42 | F=2.581 p=0.103 | 6.55 | 7.82 | 3.40 | F=2.017 p=0.162 |
| **N1** | -5.50 | -5.60 | -4.27 | F=0.633 p=0.542 | -6.39 | -7.99 | -3.61 | F=1.660 p=0.218 |
| **P2** | 9.33 | 2.37 | 4.41 | F=2.122 p=0.149 | 10.64 | 2.49 | 4.18 | F=3.00 p=0.075 |
| **N2** | -18.24 | -12.24 | -8.52 | F=2.514 p=0.109 | -20.33 | -13.59 | -9.09 | F=2.427 p=0.117 |
| **P3** | 16.06 | 17.80 | 8.78 | F=2.604 p=0.102 | 17.20 | 18.03 | 8.74 | F=1.758 p=0.201 |
| **N3** | -11.80 | -11.85 | -6.78 | F=2.743 p=0.091 | -14.24 | -11.37 | -6.42 | F=3.365 p=0.057 |

**Figure S1:** **Peak frequency of alpha/mu ERD with age in typically developing children**

**Peak frequency of alpha/mu ERD**

The individual peak frequency of alpha/mu ERD for the dominant hand was generally higher than that for the non-dominant hand (mean peak frequencies 10.41 Hz and 9.64 Hz respectively. Paired t-test: t=2.854, p=0.009). The peak frequency of alpha/mu ERD showed a trend towards increasing frequency with age for the dominant hand (Suppl Fig. S1 above), but this did not reach statistical significance (Dom hand Rho=0.379 p=0.082). There was no clear relationship for the non-dominant hand Rho=0.165, p=0.462).

**Table S9. Magnitude of beta event related desynchronisation (ERD) and synchronisation (ERS) in controls, across age-groups.** Mean levels of ERD and ERS for time windows 0.3-0.8s and 1.0-2.0s post-stimulus respectively, are shown for controls in each age-group. 95% confidence intervals are shown in brackets

|  | **Dominant hand** | | | **Non-dominant hand** | | |
| --- | --- | --- | --- | --- | --- | --- |
|  | **Controls**  **age 5-9 years** | **Controls**  **age 10-14 years** | **Controls**  **age 15-21 years** | **Controls**  **age 5-9 years** | **Controls**  **age 10-14 years** | **Controls**  **age 15-21 years** |
| **Beta ERD (%)**  Mean and 95% confidence limits | -4.89  (-8.29 to -1.49) | -4.47  (-6.87 to -2.06) | -6.22  (-11.0 to -1.40) | -4.86  (-8.05 to -1.67) | -3.66  (-7.53 to 0.21) | -3.12  (-10.45 to 4.23) |
| **Beta ERS (%)**  Mean and 95% confidence limits | 3.95  (1.37 to 6.52) | 9.59  (3.28 to 15.90) | 9.50  (0.88 to 18.12) | 3.80  (0.19 to 7.40) | 10.22  (0.67 to 19.77) | 11.85  (3.69 to 20.01) |

Mean levels of beta ERD are comparable across age-groups. (ANCOVA across age-group controlling for excursion: Dominant hand F= 0.309 p=0.739; Non-dominant hand F=2.376 p=0.125).

Mean levels of beta ERS increased slightly with age, but this difference was not statistically significant. (ANCOVA across age-group controlling for excursion: Non-dominant hand F=3.085 p=0.074; Dominant hand F=0.587 p=0.567).

**Table S10. Alpha ERD and ERS for aetiological sub-groups.**

|  | **Dominant hand** | | | | | | | | **Non-dominant hand** | | | | | | | |
| --- | --- | --- | --- | --- | --- | --- | --- | --- | --- | --- | --- | --- | --- | --- | --- | --- |
|  | **Controls**  **n=22** | **DYT1**  **n=6** | **DYT11**  **n=4** | **DYT6**  **n=2** | **KMT2B**  **n=4** | **Idiopathic**  **n=4** | **CP-HIE**  **n=6** | **CP-Ex-prem**  **n=4** | **Controls**  **n=22** | **DYT1**  **n=6** | **DYT11**  **n=4** | **DYT6**  **n=2** | **KMT2B**  **n=4** | **Idiopathic**  **n=4** | **CP-HIE**  **n=3** | **CP-Ex-prem**  **n=2** |
| **Alpha ERD** | -20.72  (-26.38- -15.05 | -9.99  (-17.30-  -2.70) | -16.87  (-40.68- 6.95) | -4.26 | -3.84  (-11.34-3.66) | -14.10  (-20.90-  -7.30 | -16.76  (-30.27-  -3.24) | -5.20  (-9.23-  -1.17) | -21.01  (-26.04-  -15.97) | -5.76  (-12.76- 1.23) | -12.37  (-21.35-  -3.39) | -13.42 | -11.78  (-22.12-  -1.44) | -6.30  (-18.96- 6.37) | -11.43  (-41.55-18.69) | 1.37 |
| **ANCOVA comparing with controls** |  | F=3.834  p=0.062 | F=0.010  p=0.921 |  | F=7.801  **p=0.011*** | F=0.887  p=0.356 | F=0.001  p=0.970 | F=6.906  **p=0.015*** |  | F=11.547  **p=0.002**** | F=0.823  p=0.374 |  | F=4.304  p=0.050 | F=5.392  **p=0.030*** | F=2.569  p=0.124 |  |
| **Alpha**  **ERS** | 15.64  (8.90-22.37) | 5.36  (-0.13-10.84) | 0.97  (-5.20-7.15) | 21.21 | 0.58  (-6.22 – 7.38) | 14.34  (-3.53-32.2) | 2.66  (-1.94-7.27) | 0.04  (-6.51 – 6.58) | 10.13  (4.91-15.35) | 8.20  (-1.21 – 17.6) | 10.14  (-1.77 – 22.05) | 12.61 | 2.81  (-3.25- 8.87) | 8.48  (-1.10 – 18.07) | 2.72  (-1.07 – 6.5) | 3.55  (-15.57 – 22.66) |
| **ANCOVA comparing with controls** |  | F=2.571  p=0.122 | F=11.654  **p=0.002**** |  | F=4.877  **p=0.038*** | F=0.399  p=0.534 | F=8.655  **p=0.007**** | F=9.234  **p=0.006**** |  | F=0.079  p=0.781 | F=0.447  p=0.511 |  | F=1.713  p=0.204 | F=1.127 p=0.301 | F=0.748 p=0.397 |  |

**Analysis of aetiological sub-groups**

As a secondary, exploratory analysis, the patterns of alpha ERD and ERS across different dystonia aetiologies were investigated. These findings are shown in Figure 8 A-B (alpha ERD) and Figure 8 C-D (alpha ERS). The small sub-group sample sizes and multiple sub-groups precludes a comparison of every combination. Rather, for this exploratory analysis, each sub-group with 4 or more participants was compared against the control group (n=22). p<0.05 was taken as significant for these secondary analyses. The small numbers mean that results will need to be confirmed in larger cohorts.

Alpha ERD:

For dominant hand movement, magnitude of alpha ERD was significantly smaller than controls in KMT2B dystonia and dystonic cerebral palsy (CP) due to prematurity (see results of statistical analysis in Table 4). For the non-dominant hand, magnitude of alpha ERD was significantly smaller than controls in DYT1 dystonia and idiopathic dystonia.

Alpha ERS:

For dominant hand movement, significantly lower levels of alpha ERS were seen in DYT11 dystonia, KMT2B dystonia, dystonic CP due to term hypoxic ischaemic encephalopathy (HIE) and dystonic CP due to prematurity (see Table 4), whereas no significant reduction was seen for DYT1 or idiopathic dystonia. For the non-dominant hand, none of the sub-groups showed a significant reduction in alpha ERS compared with controls.

**Table S11. Relationship between resting power and age**

|  | **Correlation between resting power and age** | | |
| --- | --- | --- | --- |
|  | **Controls**  N=22 | **Dystonia pre-DBS**  N=11 | **Dystonia post-DBS**  N=19 |
| **Theta 3-7Hz** | R= -0.795  p=0.000010 | R= -0.671  p=0.024 | R= -0.638  p=0.003 |
| **Alpha 8-12Hz** | R= -0.417  p=0.053 | R= -0.423  p=0.195 | R=-0.211  p=0.385 |
| **Beta 14-30Hz** | R= 0.484  p=0.023 | R=0.424  p=0.194 | R=0.429  p=0.067 |

**Table S12. Relationship between amplitude of alpha ERD/ERS and resting power**

(note alpha ERD is expressed as a negative value so a negative R value for alpha ERD actually shows that higher amplitudes of alpha resting power are associated with stronger ERD).

|  | **Correlation between ERD/ERS and resting power, controlling for age** | | |
| --- | --- | --- | --- |
|  | **Controls**  N=22 | **Dystonia pre-DBS**  N=11 | **Dystonia post-DBS**  N=19 |
| **Resting alpha and alpha ERD (non-dom hand)** | R= -0.638  p=0.002 | R= -0.304  p=0.393 | R= 0.018  p=0.954 |
| **Resting alpha and alpha ERD (dom hand)** | R= -0.521  p=0.016 | R= -0.059  p=0.871 | R= -0.172  p=0.495 |
| **Resting alpha and alpha ERS (non-dom hand)** | R= 0.428  p=0.053 | R= 0.329  p=0.353 | R=-0.300  p=0.319 |
| **Resting alpha and alpha ERS (dom hand)** | R= 0.569  p=0.007 | R=0.777  p=0.008 | R=-0.177  p=0.481 |

**Table S13. Resting power comparison between groups**

|  | **Mean Resting power (95% confidence intervals)** | | |  |
| --- | --- | --- | --- | --- |
|  | **Controls**  N=22 | **Dystonia pre-DBS**  N=11 | **Dystonia post-DBS**  N=19 | **ANCOVA**  Controlling for age |
| **Theta 3-7Hz** | 329.40  (305.65-353.15) | 362.91  (323.29-402.52)  *Genetic/Idiopathic 355.31*  *(301.07-409.55)*  *Acquired 383.16*  *(277.49-488.84)* | 230.53  (188.21-272.85)  *Genetic/Idiopathic 245.20*  *(184.05-306.36)*  *Acquired 205.38*  *(140.14-270.61)* | F(2,48)=8.010  p=0.001 |
| **Alpha 8-12Hz** | 188.33  (177.00-199.66) | 183.60  (164.69-202.51)  *Genetic/Idiopathic 184.85*  *(158.63-211.06)*  *Acquired 180.27*  *(124.82-235.73)* | 139.31  (122.03-156.59)  *Genetic/Idiopathic 142.60*  *(122.86-162.34)*  *Acquired 133.68*  *(92.03-175.32)* | F(2,48)=8.636  p=0.001 |
| **Beta 14-30Hz** | 96.03  (90.13-101.94) | 88.33  (77.14-99.52)  *Genetic/Idiopathic 92.63*  *(77.95-107.30)*  *Acquired 76.88*  *(61.45-92.30)* | 86.38  (76.93-95.84)  *Genetic/Idiopathic 81.28*  *(69.08-93.48)*  *Acquired 95.14*  *(78.15-112.12)* | F(2,48)=5.088  p=0.010 |

Comparing resting power between controls and individuals with dystonia, both pre- and post-DBS there was a significant difference in levels of theta power between groups (ANCOVA controlling for age: F (2,48)=8.010, p=0.001). Post-hoc analyses showed a trend towards higher levels of theta power in the pre-DBS group than in controls (F(1,30)=2.948, p=0.096) and significantly lower levels in the post-DBS compared with the pre-DBS group (F(1,27)=6.880, p=0.014). There were also reduced levels of alpha power in the post-DBS versus pre-DBS group ((F(1,27)=5.385, p=0.028).

**Comparison of resting power between control, genetic/idiopathic and acquired dystonia groups**

ANCOVA across groups is shown above in Table S13. Results of post-hoc analyses for each frequency band (ANCOVA, controlling for age) are as follows:

Controls vs Dystonia pre-DBS

Theta power F(1,30)=2.948 p=0.096

Alpha power F(1,30)=0.644 p=0.429

Beta power. F(1,30)=1.596 p=0.216

Controls vs Dystonia post-DBS

Theta power F(1,38)=9.525 p=0.004

Alpha power F(1,38)=16.079 p=0.000274

Beta power F(1,38)=9.736 p=0.003

Pre-DBS vs Post-DBS

Theta power F(1,27)=6.880 p=0.014

Alpha Power F(1,27)=5.385 p=0.028

Beta power F(1,27)=2.324 p=0.139
